# Do Non-climacteric Fruits Share a Common Ripening Mechanism of Hormonal Regulation?

**DOI:** 10.3389/fpls.2022.923484

**Published:** 2022-06-09

**Authors:** Dingyu Fan, Wei Wang, Qing Hao, Wensuo Jia

**Affiliations:** ^1^Institute of Horticulture Crops, Xinjiang Academy of Agricultural Sciences, Urumqi, China; ^2^College of Horticulture, China Agricultural University, Beijing, China

**Keywords:** ripening mechanism, non-climacteric fruit, ripening, hormonal regulation, crosstalk

## Abstract

Fleshy fruits have been traditionally categorized into climacteric (CL) and non-climacteric (NC) groups. CL fruits share a common ripening mechanism of hormonal regulation, i.e., the ethylene regulation, whereas whether NC fruits share a common mechanism remains controversial. Abscisic acid (ABA) has been commonly thought to be a key regulator in NC fruit ripening; however, besides ABA, many other hormones have been increasingly suggested to play crucial roles in NC fruit ripening. NC fruits vary greatly in their organ origin, constitution, and structure. Development of different organs may be different in the pattern of hormonal regulation. It has been well demonstrated that the growth and development of strawberry, the model of NC fruits, is largely controlled by a hormonal communication between the achenes and receptacle; however, not all NC fruits contain achenes. Accordingly, it is particularly important to understand whether strawberry is indeed able to represent a universal mechanism for the hormonal regulation of NC fruit ripening. In this mini-review, we summarized the recent research advance on the hormone regulation of NC ripening in relation to fruit organ origination, constitution, and structure, whereby analyzing and discussing whether NC fruits may share a common mechanism of hormonal regulation.

## Introduction

Based on the physiological behaviors of fruit ripening, fleshy fruits have been traditionally categorized into climacteric (CL) and non-climacteric groups (NC). CL fruits are characterized by a burst of respiration and ethylene production at the onset of fruit ripening, whereas NC fruits do not show such characteristics (Giovannoni, 2001; Cherian et al., 2014; Giovannoni et al., 2017; Fenn and Giovannoni, 2021; Kou et al., 2021; Li et al., 2021). It has been well established that CL fruits share a common mechanism of ripening regulation, i.e., the ethylene regulation (Alexander and Grierson, 2002; Liu et al., 2015; Brumos, 2021; Li et al., 2021). This conclusion has been drawn not only based on that fact CL fruits show a burst of ethylene production at the onset of fruit ripening, but also on the fact that application of exogenous ethylene is capable of sensitively triggering CL fruit ripening, and conversely, inhibiting the ethylene action is capable of effectively arresting CL fruit ripening (Fray and Grierson, 1993; Alexander and Grierson, 2002; Barry and Giovannoni, 2007; Gapper et al., 2013; Li et al., 2021). The theory of ethylene regulation has been commercially exploited to control the shelf-life of CL fruits, undoubtedly demonstrating the powerful role of ethylene in CL fruit ripening.

In recent years, there has been a growing interest in the mechanistic studies on NC fruit ripening, and strawberry emerges to become a model for study on NC fruit ripening (Cherian et al., 2014; Kuhn et al., 2014; Moya-León et al., 2019; Alferez et al., 2021). While abscisic acid (ABA) has been proposed to play an important role in NC fruit ripening (Kumar et al., 2014; Forlani et al., 2019; Fenn and Giovannoni, 2021; Kou et al., 2021; Li et al., 2021), there are evidence that many other hormones may also play crucial roles in NC fruit ripening. For example, auxin (Given et al., 1988; Gu et al., 2019; Vincent et al., 2020; Castro et al., 2021; Clayton-Cuch et al., 2021; Li et al., 2022), giberelin (Kou et al., 2021), jasmonic acid (Creelman and Mullet, 1997; Concha et al., 2013; Preuß et al., 2014; Jia et al., 2016; Coelho et al., 2019; Li et al., 2022), Brassinosteroids (Symons et al., 2006), and even ethylene (Dong et al., 2020; Figueroa et al., 2021), all these hormones have been reported to be involved in the regulation of strawberry fruit ripening. Importantly, as early as 1980s, a classic study by Given et al. (1988) demonstrated that removal of achenes from receptacle induced fruit ripening, and it was concluded that a decrease in the level of auxin (IAA) in receptacle might act as a signal regulating strawberry fruit ripening. Notably, the IAA signal is so strong that removal of the achenes could quickly induce strawberry fruit pigmentation, and conversely, application of IAA to receptacle surface could prevent the achenes’ removal-induced pigmentation. By contrast, while ABA has been commonly regarded as the key regulator controlling NC fruit ripening, there are reports that induction of ABA directly to receptacle *via* injection was not found to significant affect fruit ripening (Symons et al., 2012; Li et al., 2022). Therefore, whether ABA might be indeed the key regulator controlling strawberry fruit ripening is doubtful.

As both IAA and ABA have been suggested to play crucial roles in strawberry fruit ripening, in a recent study, we investigated a potential mechanism for a synergistic action between ABA and IAA in strawberry fruit ripening. We found that ABA could modulate IAA transport from achenes to receptacle, such that ABA and IAA might jointly regulate strawberry fruit ripening. This investigation also suggested that NC fruit ripening might be correlated with fruit constitution and structure. NC fruits vary greatly in their origin, constitution, and structure (Liu et al., 2020). For example, while some NC fruits belong to true fruit (i.e., derived from ovary), such as Bilberry (*Vaccinium myrtillus*), Blackberry (*Rubus fruticosus*), and Grape berry (*Vitis vinifera*), some others belong to spurious/false fruits (i.e., derived from a combined structure of ovary with other tissues), such as, Cashew-apple (*Anacardium occidentable*), Gooseberry (*Phyllanthus acidus*), and Strawberry (*Fragaria ananassa*). While NC fruits have been traditionally regarded as one group, and therefore, proposed to share a common mechanism of fruit ripening, little attention has been paid to the question of whether the hormonal regulation of NC fruit ripening might be correlated to fruit origin and structure. In this mini-review, we summarized the recent research advance in the hormonal regulation of NC fruit ripening in relation to fruit origination, constitution, and structure, whereby analyzing and discussing whether NC fruits may share a common mechanism of fruit ripening.

## Features of NC Fruit Ripening in Relation to Fruit Structure

Based on fruit origin, fruits can be divided into true and spurious/false fruits. True fruits are originated from ovary, such as bilberry (*V. myrtillus*), blackberry (*R. fruticosus*), and grape berry (*V. vinifera*), and false fruits are originated from a combined structure of ovary with other tissues, such as strawberry (*Fragaria ananassa*), pineapple (*Ananas comosus*), cashew-apple (*Anacardium occidentable*), and gooseberry (*P. acidus*). False fruits again vary greatly in structure. For example, strawberry is derived from a combination of receptacle and ovaries that ultimately form achenes, whereas pineapple is formed from a combination of inflorescence axes. Based on fruit constitution, fruits can be divided into simple fruits (i.e., derived from a sole ovary), aggregate fruits (i.e., derived from a collection of multiple ovaries), and collective fruits (i.e., derived from a collection of multiple flowers). In addition, based on the texture and anatomical features of ripened fruit, fruits can be also classified into berry fruit, stone fruit, pome fruit, hesperdium fruit, litchi fruit, etc. Even for berry fruits, their textures may be quite different depending on species, cultivars, and genotypes. For example, while some berry fruits are very succulent, delicate, and soft, such as blackberry (*R. fruticosus*), mulberry (*Fructus Mori*), raspberry (*Rubus corchorifolius*), and strawberry (*Fragaria ananassa*), the texture of some other berry fruits may be relatively firmer with less juice, such as, avocado (*Persea Americana*), banana (*Musa spp.*), and papaya (*Carica papaya*). Plant morphogenesis is determined by a synergistic action of multiple hormones and different organizational structures may be different in their specific pattern of the synergistic action (Savidge, 1983; Schiefelbein, 1994). For example, while shoot morphogenesis is mainly determined by a synergistic action of IAA, gibberellin, brassinolide, etc. (Fleming, 2005), root morphogenesis is determined by cytokinin, ABA, IAA, etc. (Schiefelbein et al., 1997). Based on the complexity of fruit structure, it is reasonable to doubt that different types of NC fruits share a common mechanism for their ripening regulation.

**Figure 1:**
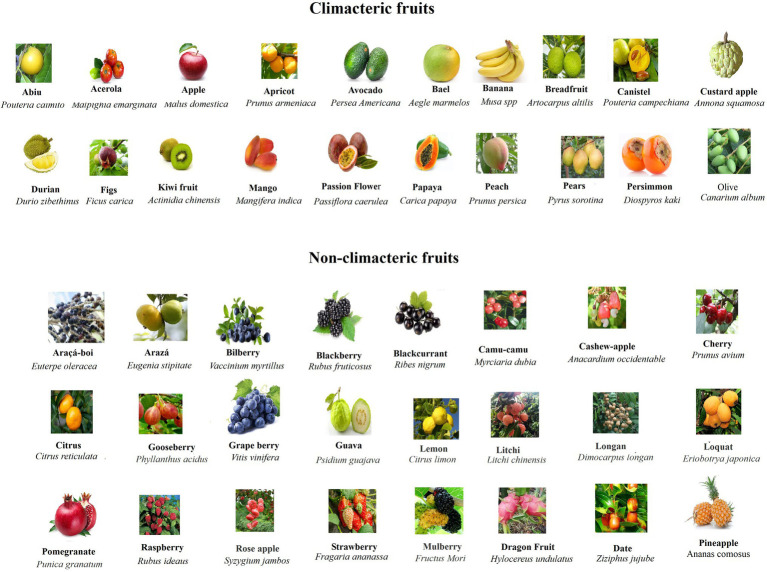
Pictures of typical CL and NC fruits showing the appearance features of different fruits. Note that NC fruits are commonly more colorful and their sizes are commonly smaller in comparison with CL fruits.

In order to analyze and compare the ripening behavior of different fruits in relation to their structures, we collected and sorted out a series of typical fruits in both CL and NC group. As shown in Figure 1 and Table 1, the CL and NC classes both contain true and false fruits, and moreover, both contain simple, aggregate, and collective fruits. For example, while some true fruits, such as bilberry (*V. myrtillus*), blackberry (*R. fruticosus*), and citrus (*Citrus reticulata*), belong to NC fruits, some other true fruits, such as avocado (*Persea Americana*), kiwi fruit (*Actinidia chinensis*), and peach (*Prunus persica*), belong to CL fruits. Likewise, while some false fruits, such as araçá-boi (Eugenia stipitate), camu-camu (Myrciaria dubia), and raspberry (Rubus corchorifolius), belong to NC fruits, some other false fruits, such as apple (*Malus domestica*), banana (Musa spp), and pears (Pyrus sorotina), belong to CL fruits. In addition, regardless of simple, collective, or aggregate fruits, they are all included in the NC and CL classes. Information above suggests that the behaviors of fruit ripening are not correlative to their origin or constitution. However, when fruit texture is considered, it appears that nearly all NC fruits exhibit a succulent and soft texture, which is distinctly different from that of CL fruits (Table 1). This implies that the ripening behavior of fruit ripening is likely correlative to the changing pattern of fruit structure, regardless of fruit origin and constitution. Nevertheless, there are several exceptions, in which a few of NC fruits show distinct texture from most of the NC fruits, such as dragon Fruit (*Hylocereus undulatus*), date (*Ziziphus jujuba*), and pineapple (*A. comosus*). Although these fruits do not belong to berry fruits, they do show a similar ripening behavior, i.e., the onset feature of their ripening is not quite stringent. These observations suggest a necessity of a finer classification for NC fruits.

**Table 1:** Comparison of fruit origin, structure, and ripening behaviors between CL and NC fruits.

Fruit name	Fruit origin and structure		Texture of ripen fruit		Ripening behavior
	True or false fruit	Simple fruit or not	Berry or not	Juicy or not	
Araçá-boi (*Euterpe oleracea*)	True	Yes	Yes	Yes	NC
Araza (*Eugenia stipitate*)	True	No	Yes	Yes	NC
Bilberry (*Vaccinium myrtillus*)	True	Yes	Yes	Yes	NC
Blackberry (*Rubus fruticosus*)	True	Yes	Yes	Yes	NC
Blackcurrant (*Ribes nigrum*)	True	No	Yes	Yes	NC
Camu-camu (*Myrciaria dubia*)	False	Yes	Yes	Yes	NC
Cashew-apple (*Anacardium occidentable*)	False	Yes	Yes	Yes	NC
Cherry (*Prunus avium*)	True	Yes	Yes	Yes	NC
Citrus (*Citrus reticulata*)	True	Yes	Yes	Yes	NC
Gooseberry (*Phyllanthus acidus*)	False	Yes	Yes	Yes	NC
Grape berry (*Vitis vinifera*)	True	Yes	Yes	Yes	NC
Guava (*Psidium guajava*)	False	Yes	Yes	Yes	NC
Lemon (*Citrus limon*)	True	Yes	Yes	Yes	NC
Litchi (*Litchi chinensis*)	True	Yes	Yes	Yes	NC
Longan (*Dimocarpus longan*)	True	Yes	Yes	Yes	NC
Loquat (*Eriobotrya japonica*)	False	Yes	Yes	Yes	NC
Mulberry (*Fructus Mori*)	True	No	Yes	Yes	NC
Pomegranate (*Punica granatum*)	False	Yes	Yes	Yes	NC
Raspberry (*Rubus ideaus*)	False	No	Yes	Yes	NC
Rose apple (*Syzygium jambos*)	False	Yes	Yes	Yes	NC
Strawberry (*Fragaria ananassa*)	False	No	Yes	Yes	NC
Dragon Fruit (*Hylocereus undulatus*)	False	Yes	No	No	NC
Date (*Ziziphus jujube*)	True	Yes	No	No	NC
Pineapple (*Ananas comosus*)	False	No	No	No	NC
Abiu (*Pouteria caimito*)	True	Yes	Yes	No	CL
Acerola (*Malpighia emarginata*)	True	Yes	Yes	No	CL
Apple (*Malus domestica*)	False	Yes	No	No	CL
Apricot (*Prunus armeniaca*)	True	Yes	No	No	CL
Avocado (*Persea Americana*)	True	Yes	Yes	No	CL
Bael (*Aegle marmelos*)	True	Yes	No	No	CL
Banana (*Musa spp*)	False	Yes	Yes	No	CL
Breadfruit (*Artocarpus altilis*)	True	No	No	No	CL
Canistel (*Pouteria campechiana*)	True	Yes	Yes	No	CL
Custard apple (*Annona squamosa*)	True	No	Yes	No	CL
Durian (*Durio zibethinus*)	False	Yes	Yes	No	CL
Figs (*Ficus carica*)	False	Yes	Yes	No	CL
Kiwi fruit (*Actinidia chinensis*)	True	Yes	Yes	No	CL
Mango (*Mangifera indica*)	True	Yes	Yes	No	CL
Passion Flower (*Passiflora caerulea*)	True	Yes	Yes	No	CL
Papaya (*Carica papaya*)	True	Yes	Yes	No	CL
Peach (*Prunus persica*)	True	Yes	No	No	CL
Pears (*Pyrus sorotina*)	False	Yes	No	No	CL
Persimmon (*Diospyros kaki*)	True	Yes	Yes	No	CL

Note that no matter what types of fruits, i.e. true or false fruits as well as simple or aggregate fruits, they are all included in CL and NC fruits, and also note that while nearly all the juicy and soft fruits are included in NC group, no juicy and soft fruits are included in CL group.

In nature, fruit production aims to disperse seeds, so that plants can survive and thrive in ever-changing environment. Ripening of fleshy fruits involves dramatic changes in cell physiology and biochemical metabolism, such as color, sugar, acid, aroma, and texture, whereby attracting animals to disperse seeds (Fukano and Tachiki, 2021). Theoretically, different ripening behavior may be an adaptation to seeds dispersers. Indeed, CL fruits were commonly found to ripen after release from parent plant, whereas NC fruits ripen only on the three. The ripening behavior after release from the parent plant for CL fruits has been proposed to be an adaptation to ground dispersers of some large herbivorous and omnivorous mammals, whereas NC fruits have been proposed to be an adaptation to tree dispersers, such as birds, bats, and primates (Eriksson et al., 2000; Gao et al., 2019; Fukano and Tachiki, 2021). As mentioned above, the ripening behavior of NC fruits is likely correlative to their structural bases, i.e., their texture is much succulent and soft. From an evolutional point of view, the succulent and soft texture means that, once falling down on the ground, they will quickly become rotten, and this is obviously not in favor of their widespread seeds’ dispersing. By contrast, tree dispersers are more likely to disperse seeds distantly and this may be why NC fruits commonly ripen on tree. Besides fruit texture, NC fruits appear to exhibit some other common features, such as bright color and smaller size (Figure 1). Bright color is known to more likely attract birds, bats, and the three dispersers, and smaller size is obviously in favor of being consumed by birds and bats. Obviously, all these features may be an adaption to seeds’ dispersing.

## Hormonal Regulation of NC Fruit Ripening

Early studies on hormonal regulation of NC fruit ripening were mainly based on pharmacological experiments. Unlike the regulation of CL fruit ripening with ethylene as a major regulator, nearly all hormones have been reported to play an important role in NC fruit ripening (Given et al., 1988; Creelman and Mullet, 1997; Symons et al., 2006; Concha et al., 2013; Preuß et al., 2014; Jia et al., 2016; Coelho et al., 2019; Gu et al., 2019; Dong et al., 2020; Vincent et al., 2020; Castro et al., 2021; Clayton-Cuch et al., 2021; Figueroa et al., 2021; Kou et al., 2021; Li et al., 2022). To date, it is hard to conclude which hormone may be the key regulator controlling NC fruit ripening.

As early as 1980s, a classic study by Given et al. (1988) demonstrated that removal of achenes from receptacle induced strawberry fruit ripening (Given et al., 1988). Since IAA is synthesized in achenes (Given et al., 1988; Liu et al., 2020), it was proposed that a decrease in the level of IAA may serve as a signal controlling strawberry fruit ripening. Later studies *via* molecular techniques provided more direct evidences that IAA plays a pivotal role in strawberry fruit ripening. For example, it was demonstrated that over-expression of *FaAux/IAA*, the transcription factor in the IAA signaling pathway, as well as over-expression of *FaARF*, an IAA response factors, delayed the onset of strawberry ripening (Given et al., 1988; Liu et al., 2011). Similarly, it was reported that application of IAA or NAA (the synthetic auxin, 1-naphthaleneacetic acid) delayed grape berry ripening (Coombe and Hale, 1973; Böttcher et al., 2012; Ziliotto et al., 2012; Davies et al., 2015). Furthermore, it was found that application of NAA to pre-veraison grape berries resulted in a significant change in the expression of many ripening-associated genes (Dal Santo et al., 2020).

In recent years, particular attention has been paid to the role of ABA in NC fruit ripening (Aharoni and O'Connell, 2002; Fenn and Giovannoni, 2021). In grape, it was reported that application of exogenous ABA could induce an accumulation of anthocyanins, flavonols, resveratrol, and other ripening-associated compounds (Koyama et al., 2010; Wang et al., 2016; Forlani et al., 2019; Zhang et al., 2021). Molecular study further demonstrated that over-expression of *VvABF2*, an ABA responsive transcription factor, in grapevine cell suspensions induced an accumulation of some ripening-associated compounds (Zhu et al., 2016). In strawberry, it was reported that as ripening proceeds, ABA levels progressively increase and peak at the fully ripe stage (Jia et al., 2011; Symons et al., 2012; Li et al., 2015; Kim et al., 2019). By using virus-induced gene silencing (VIGS), it was reported that downregulation of *FaNCED1*, a key gene in the ABA synthesis pathway, or *FaCHLH/ABAR*, encoding a putative ABA receptor, arrested ripening (Chai et al., 2011; Jia et al., 2011, 2013). However, it was later reported that there are some arguments about the use of VIGS in strawberry, because it may inherently perturb ripening (Zhao et al., 2019). So far, there is no direct evidence, i.e., directly manipulating the ABA action *via* stably transgenic strategy, supporting a major role of ABA in NC fruit ripening.

In addition to IAA and ABA, there is evidence that jasmonic acid (JA) may also play an important role in NC fruit ripening. In grape berry, a number of studies demonstrated that application of MeJA (Jasmonic acid methyl ester) promoted accumulation of a variety of ripening-associated compounds, such as phenolic compounds (Ruiz-García et al., 2012, 2013; Rocio, 2017), anthocyanins (Jia et al., 2016; Paladines-Quezada et al., 2021), volatile compounds (Garrido-Bigotes et al., 2018b), and resveratrol (Vezzulli et al., 2007). In strawberry, it was reported that application of MeJA promoted the accumulation of anthocyanins (Pérez et al., 1997; Concha et al., 2013; Delgado et al., 2018; Garrido-Bigotes et al., 2018a,b) and aroma-associated compounds (Moreno Fde et al., 2010). There is evidence that JA may function in a synergistic manner with ABA in strawberry fruit ripening. For example, a study reported that application of exogenous MeJA caused a decrease in ABA content and altered the expression profile of several cell wall metabolism-associated genes. Moreover, transiently manipulating the expression of FvJAZs, the key signal component in the JA signaling pathway delayed strawberry fruit ripening, suggesting that JA may be an important signal regulating NC fruit ripening (Garrido-Bigotes et al., 2018b).

**Figure 2:**
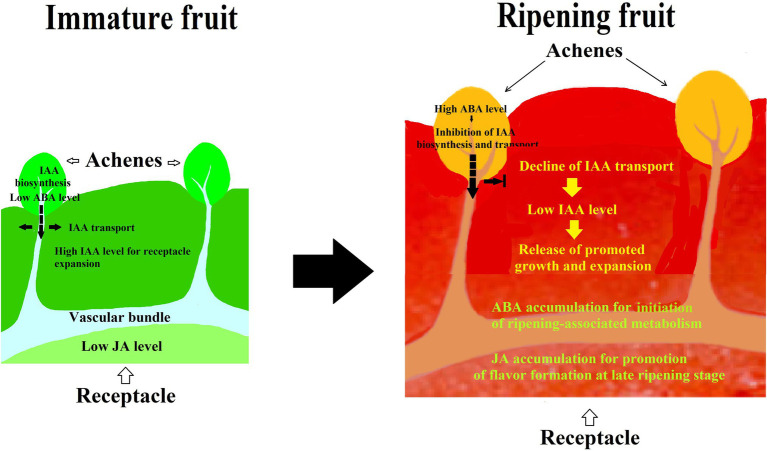
Diagram showing the pattern of hormonal regulation of strawberry fruit ripening. IAA is synthesized in achenes and then transported to the receptacle, whereby promoting receptacle growth and suppressing fruit ripening. Along with fruit development and ripening, ABA accumulates in both achenes and receptacle. ABA accumulation in the achenes acts to suppress the IAA biosynthesis and transport to the receptacle, resulting in a decrease in the IAA level of the receptacle and release of the ripening inhibition. ABA accumulation in the receptacle acts to initiate ripening-associated metabolisms, and JA accumulation may likely act to regulate the ripening-associated events at a later ripening stage. As such, strawberry fruit ripening is regulated by a synergistic action of multiple hormones.

Given the fact that many hormones have been suggested to be involved in strawberry fruit ripening, there is a possibility that strawberry fruit ripening is regulated by a synergistic action of different hormones, rather than by a sole hormone. To demonstrate such a hypothesis, we investigated the synergistic action among IAA, ABA, and JA in strawberry fruit ripening. In a pharmacological experiment, we found that the effect of ABA was affected by its application method, i.e., while injecting ABA directly into the receptacle was not found to have perceptible effect on fruit ripening, feeding ABA *via* fruit pedicel indeed significantly promoted ripening. Moreover, the results showed that ABA was able to modulate an IAA transport from the achenes to receptacle, implying that strawberry fruit ripening is regulated by a synergistic action between ABA and IAA (Li et al., 2022). Surprisingly, while ABA application to receptacle *via* injection was not found to significantly affect strawberry fruit ripening, MeJA application was found to be capable of significantly promoting fruit ripening, indicating that JA may be an important signal in strawberry fruit ripening (Li et al., 2022). Collectively, these observations strongly suggest that strawberry fruit ripening is regulated by a synergistic action of multiple hormones (Figure 2).

Strawberry has been traditionally thought to be a typical NC fruit and therefore regarded as a model plant for studies on NC fruit ripening. As discussed above, strawberry fruit ripening is largely regulated by a hormonal communication between achenes and receptacle. Since not all NC fruits consist of achenes and receptacle, it is doubtful whether ripening mechanism of strawberry commonly exists in all NC fruits. Strawberry and raspberry both belong to aggregated fruit derived from numerous ovaries and receptacle. However, while the edible part of strawberry is fleshy receptacle, the edible part of raspberry largely consists of numerous fleshy drupes derived from ovaries. Accordingly, a comparison study between strawberry and raspberry would expect to provide valuable information on the mechanism of NC fruit ripening. A study by Moro et al. (2017) showed that raspberry fruit ripening could be inhibited by IAA and promoted by JA. Moreover, a number of studies reported that calcium signaling may play an important role in the regulation of strawberry fruit ripening (Xu et al., 2014; Chen et al., 2016; Peng et al., 2016). Similarly, Kayal et al. (2017) reported that calcium signaling is involved in raspberry fruit ripening. Overall, while it could not be concluded that NC fruits share a common mechanism, it appears that aggregate NC fruits may likely share a similar mechanism of fruit ripening.

## Conclusion and Future Perspectives

Given that ethylene has been well demonstrated to be a key regulator controlling CL fruit ripening, identification of the key regulator controlling NC fruit ripening has been a major interest in the studies on NC fruit ripening. However, to date, what may act as the key regulator is still a question of controversial. In the past years, it has been increasingly suggested that ABA may be a key regulator in NC fruit ripening. Logically, as a key regulator, its application should be able to strongly modulate NC fruit ripening. However, in the NC model of strawberry, while ABA has been well established to be involved in fruit ripening, there is no report that application of ABA to receptacle *via* injection was capable of remarkably promoting fruit ripening (Symons et al., 2012; Li et al., 2022). More importantly, our resent study demonstrated that it is a synergic action of ABA and IAA, alternatively the ABA-modulated IAA translocation from achenes to receptacle that plays a crucial role in the regulation of strawberry fruit ripening (Li et al., 2022). Because not all NC fruits consist of receptacle and achenes, it is reasonable to propose that NC fruits should not share a common mechanism of hormonal regulation. To profoundly elucidate the mechanism of NC fruit ripening, several aspects should be researched with priority as follows:

### Synergistic Action Among Different Hormones in NC Fruit Ripening

Since ethylene has been established to be a major hormone controlling CL fruit ripening, there is a growing interest in the identification of the correspondingly major hormone in NC fruit ripening. While ABA has been commonly thought to be an important hormone in NC fruit ripening, it is not conclusive that it is just the major hormone controlling NC fruit ripening, as like ethylene controlling CL fruit ripening. Supposing that NC fruits may not share a common mechanism of hormonal regulation, an attempt to identify a major hormone controlling NC fruit ripening would likely be in vain. In strawberry, it has been demonstrated that fruit ripening is controlled by a synergistic action of multiple hormones (Li et al., 2022). Accordingly, to unravel the mechanism of NC fruit ripening, more attention should be paid to the mechanism for a synergistic action of different hormones in NC fruit ripening.

### Molecular Manipulation of Hormone Action in NC Fruit Ripening

In CL fruit ripening, the conclusion for ethylene to be a major signal has been drawn mainly based on three lines of evidences. Firstly, there is a burst of ethylene production at the onset of CL fruit ripening; secondly, application of exogenous ethylene is able to sensitively trigger CL fruit ripening; and thirdly, molecular manipulation of the ethylene action is able to effectively modulate CL fruit ripening. In tomato plant, for example, mutation of the ethylene receptor (such as the Nr mutant) has been demonstrated to cause a never-ripening phenotype (Alexander and Grierson, 2002; Fenn and Giovannoni, 2021). To date, while many hormones have been reported to play important roles in NC fruit ripening, the study on a manipulation of the hormone action *via* stable transgenic strategy has been lacking. Given that the stable transgenic technology, particularly the CRISPR/Cas9 technique, has been well established in strawberry, the model of NC fruits (Liu et al., 2020), studies on molecular manipulation of the key genes in hormone biosynthesis or signal transduction would expect to provide conclusive evidence for a specific role of different hormones in NC fruit ripening.

### Finer Classification of NC Fruits

Fleshy fruits have been traditionally classified into CL and NC groups; however, such a classification may be an oversimplification because some NC fruits may show, to some extent, a climacteric behavior, vice versa (Barry and Giovannoni, 2007; Obando et al., 2007). Moreover, both NC fruits’ structure and ripening behaviors vary very much depending on species and cultivars. Accordingly, to unravel the mechanism for NC fruit ripening, it is important to carry out a finer classification of the NC fruits, based on a collective consideration of respiration, ethylene production, rate of ripening progress, and the pattern of changes, etc.

### Determination of the Key Event in NC Fruit Ripening

Fruit ripening involves dramatic changes in a series of physiological and biochemical events, such as color, sugar, acids, aroma, and firmness. In CL fruits, these events happen together once the fruit ripening is initiated. Unlike CL fruits, NC fruits ripen slowly on tree, and for some species, even without a clear sign of ripening onset. Accordingly, to elucidate the mechanism for NC fruit ripening, characterization of the key event is important. Change of color has been generally selected as the maker event in fruit ripening. However, fruit pigmentation does not definitely affect fruit ripening, as evidenced by that fact that a color mutation of strawberry (e.g., Herb of yellow hairy strawberry) does not affect normal ripening. In a recent study, we demonstrated that the change of fruit firmness is essentially determined by cell wall degradation, which is again determined by the cell separation initiated at a very early stage of the fruit development (Zhang et al., 2020). Since cell wall degradation would inevitable produce profound impacts on cellular signal transduction, it is reasonable to propose that the decrease in fruit firmness should be a better marker event in NC fruit ripening.

## Author Contributions

WJ designed the paper. WJ and QH drafted the manuscript. DF and WW prepared the data and materials for the paper. All authors contributed to the article and approved the submitted version.

## Funding

This work is financially supported by the National Natural Science Foundation of China (31872086), the National Key Research and Development Program (2019YFD1000204 and 2019YFD1000803), the Beijing Science and Technology Innovation and Service Capacity in Top Subjects (CEFF-PXM2019_014207_000032), and Project of Renovation Capacity Building for the Young Sci-Tech Talents Sponsored by Xinjiang Academy of Agricultural Sciences (xjnkq-2020001).

## Conflict of Interest

The authors declare that the research was conducted in the absence of any commercial or financial relationships that could be construed as a potential conflict of interest.

## Publisher’s Note

All claims expressed in this article are solely those of the authors and do not necessarily represent those of their affiliated organizations, or those of the publisher, the editors and the reviewers. Any product that may be evaluated in this article, or claim that may be made by its manufacturer, is not guaranteed or endorsed by the publisher.
